# Supplementary material for: Seaweeds of the Israeli Mediterranean Sea: Nutritional and Biotechnological Potential Through Seasonal and Species Variation
Source: Mar Drugs. 2025 Aug 4;23(8):320. doi: 10.3390/md23080320 (PMC12387171; doi:10.3390/md23080320)
Supplement: Supplementary file 1 [file marinedrugs-23-00320-s001.zip › Table S1 Seaweeds seasonal checklist and Table S2 Statistical summary.pdf]

Supplementary Materials

Table S1. Seaweeds seasonal checklist.

	Species name	2018-2019				2019-2020					
		Spring	Summer	Autumn	Winter	Spring	Summer	Autumn	Winter	Spring	Summer
**1**	*Acanthophora najadiformis*		✓	✓	✓		✓	✓		✓	
**2**	*Alsidium corallinum*	✓	✓		✓			✓			
**3**	*Asparagopsis taxiformis*		✓		✓			✓			✓
**4**	*Bryopsis mucosa*				✓	✓					
**5**	*Bryopsis plumosa*				✓				✓		
**6**	*Caulerpa mexicana*		✓	✓							
**7**	*Chondracanthus acicularis*	✓				✓	✓		✓	✓	
**8**	*Chondria dasyphylla*		✓								
**9**	*Cladophora pellucida*	✓		✓							
**10**	*Cladophora pseudopellucida*							✓			
**11**	*Cladophoropsis* [illegible]			✓				✓			
**12**	*Codium adhaerens*	✓	✓	✓	✓	✓	✓		✓		✓
**13**	*Codium parvulum*						✓		✓		
**14**	*Colpomenia sinuosa*	✓			✓	✓				✓	
**15**	*Corallina elongata*	✓	✓	✓	✓	✓	✓	✓	✓	✓	
**16**	*Cystoseira compressa*				✓						
**17**	*Gongolaria rayssiae*	✓			✓	✓	✓				✓
**18**	*Dasya* sp.				✓	✓			✓		
**19**	*Dictyota* sp.	✓									
**20**	*Dictyota dichotoma*	✓	✓		✓	✓	✓	✓	✓	✓	✓
**21**	*Digenea simplex*	✓	✓	✓	✓						✓
**22**	*Ectocarpus siliculosus*	✓	✓		✓						
**23**	*Galaxaura rugosa*	✓	✓	✓	✓			✓	✓		
**24**	*Gelidium pusillum*	✓	✓	✓	✓	✓	✓	✓	✓	✓	
**25**	*Gelidium crinale*				✓						
**26**	*Gracilaria bursa-pastoris*	✓			✓	✓					
**27**	*Gracilaria conferta*	✓			✓		✓	✓			
**28**	*Gracilaria cornea*									✓	
**29**	*Gracilaria* sp.					✓					
**30**	*Grateloupia* sp.				✓					✓	
**31**	*Halopteris scoparia*	✓	✓			✓	✓				✓
**32**	*Halymenia dichotoma*						✓				
**33**	*Hypnea musciformis*	✓	✓		✓	✓	✓	✓		✓	
**34**	*Hypnea* sp.	✓									

**35**	*Jania rubens*	✓	✓	✓	✓	✓	✓	✓	✓	✓	✓
**36**	*Laurencia papillosa*	✓	✓	✓	✓	✓	✓	✓	✓	✓	✓
**37**	*Laurenciella marilzae*	✓	✓	✓	✓				✓		✓
**38**	*Liagora* sp.	✓	✓								
**39**	*Lobophora variegata*	✓					✓				
**40**	*Nemalion helminthoides*	✓	✓		✓	✓				✓	
**41**	*Padina pavonica*	✓	✓	✓	✓	✓	✓	✓	✓	✓	✓
**42**	*Neopyropia elongata*				✓					✓	
**43**	*Pterocladiella capillacea*	✓				✓	✓		✓	✓	
**44**	*Rytiphlaea tinctoria*							✓			
**45**	*Sargassum vulgare*	✓			✓	✓			✓	✓	✓
**46**	*Scytosiphon lomentaria*				✓				✓		
**47**	*Solieria filiformis*									✓	
**48**	*Spatoglossum solieri*									✓	
**49**	*Spyridia filamentosa*				✓	✓	✓			✓	✓
**50**	*Stypopodium schimperi*	✓				✓					✓
**51**	*Taonia atomaria*			✓							
**52**	*Ulva californica*		✓								
**53**	*Ulva compressa*	✓	✓		✓	✓		✓	✓	✓	
**54**	*Ulva fasciata*	✓	✓		✓					✓	✓
**55**	*Ulva rigida*	✓	✓		✓	✓	✓	✓	✓	✓	

Table S2. Statistical summary.

Protein (%)

**Species**	**Comparison**	***df***	**Sum Sq**	**Mean Sq**	***F***	***p* Value**	**Test**
Laurencia	Season	3	93.87	31.29	20.28	<0.0001	One-way Anova
	Residuals	17	26.22	1.54			
			**difference**	**lwr**	**upr**		
Laurencia post-hoc	Spring-Autumn		-1.43	-3.80	0.94	0.35	TukeyHSD
	Summer-Autumn		-2.20	-4.48	0.08	0.06	TukeyHSD
	Winter-Autumn		3.02	0.74	5.30	<0.01	TukeyHSD
	Summer-Spring		-0.77	-2.91	1.37	0.74	TukeyHSD
	Winter-Spring		4.45	2.32	6.59	<0.0001	TukeyHSD
	Winter-Summer		5.22	3.18	7.26	<0.0001	TukeyHSD
	**Comparison**	***df***	**Sum Sq**	**Mean Sq**	***F***	***p* Value**	**Test**
Jania	Season	3	14.79	4.93	6.92	<0.01	One-way Anova
	Residuals	18	12.82	0.71			
			**difference**	**lwr**	**upr**		

Gracilaria post-hoc	Spring-Autumn		0.04	-1.33	1.42	0.99	TukeyHSD
	Summer-Autumn		-0.80	-2.18	0.58	0.38	TukeyHSD
	Winter-Autumn		1.67	0.13	3.21	0.03	TukeyHSD
	Summer-Spring		-0.84	-2.22	0.53	0.34	TukeyHSD
	Winter-Spring		1.63	0.09	3.17	0.04	TukeyHSD
	Winter-Summer		2.47	0.93	4.01	<0.01	TukeyHSD
	**Comparison**	***df***	**Sum Sq**	**Mean Sq**	***F***	***p* Value**	**Test**
Hypnea	Season	3	10.66	3.55	1.356	0.32	Permutation Anova
	Residuals	22	57.66	2.62			
	**Comparison**	***df***	**Sum Sq**	**Mean Sq**	***F***	***p* Value**	**Test**
Padina	Season	3	32.86	10.95	4.88	<0.01	One-way Anova
	Residuals	25	56.10	2.24			
			**difference**	**lwr**	**upr**		
Padina post-hoc	Spring-Autumn		-0.73	-3.25	1.80	0.86	TukeyHSD
	Summer-Autumn		-0.24	-2.72	2.23	0.99	TukeyHSD
	Winter-Autumn		1.94	-0.59	4.46	0.18	TukeyHSD
	Summer-Spring		0.49	-1.52	2.49	0.91	TukeyHSD
	Winter-Spring		2.67	0.60	4.73	0.01	TukeyHSD
	Winter-Summer		2.18	0.18	4.18	0.03	TukeyHSD
	**Comparison**	***df***	**Sum Sq**	**Mean Sq**	***F***	***p* Value**	**Test**
Sargassum	Season	2	47.08	23.54	7.93	<0.01	Permutation Anova
	Residuals	17	50.48	2.97			
			**t**	**difference**			
Sargassum post-hoc	Summer-Spring		0.42	5.01		0.90	Games-Howell
	Winter-Spring		2.45	5.39		0.12	Games-Howell
	Winter-Summer		14.64	11.63		<0.0001	Games-Howell
	**Comparison**	***df***	**Sum Sq**	**Mean Sq**	***F***	***p* Value**	**Test**
Dictyota	Season	3	166.73	55.58	116.6	<0.0001	Permutation Anova
	Residuals	20	9.53	0.48			
			**t**	**difference**			
Dictyota post-hoc	Spring-Autumn		2.44	5.41		0.18	Games-Howell
	Summer-Autumn		6.10	5.55		<0.01	Games-Howell
	Winter-Autumn		20.03	8.70		<0.0001	Games-Howell
	Summer-Spring		2.19	9.77		0.19	Games-Howell
	Winter-Spring		12.43	7.92		<0.0001	Games-Howell

	Winter-Summer		16.77	8.84		<0.0001	Games-Howell
	**Comparison**	***df***	**Sum Sq**	**Mean Sq**	***F***	***p* Value**	**Test**
Ulva	Season	3	82.89	27.63	7.289	<0.01	Permutation Anova
	Residuals	19	72.02	3.79			
			**t**	**difference**			
Ulva post-hoc	Spring-Autumn		2.24	3.98		0.26	Games-Howell
	Summer-Autumn		0.11	8.89		0.99	Games-Howell
	Winter-Autumn		6.89	6.83		<0.01	Games-Howell
	Summer-Spring		1.11	10.88		0.69	Games-Howell
	Winter-Spring		7.25	7.66		<0.001	Games-Howell
	Winter-Summer		3.60	11.48		0.02	Games-Howell
	**Comparison**	***df***	**Sum Sq**	**Mean Sq**	***F***	***p* Value**	**Test**
Codium	Season	3	0.88	0.29	0.90	0.4863	One-way Anova
	Residuals	17	5.53	0.33			
Protein species comparison	**Comparison**	***df***	**Sum Sq**	**Mean Sq**	***F***	***p* Value**	**Test**
	Seaweed species	7	1064.92	152.13	36.59	<0.0001	Permutation Anova
	Residuals	178	740.12	4.16			

Antioxidant activity (mg TE g^{-1} DW)

**Species**	**Comparison**	***df***	**Sum Sq**	**Mean Sq**	***F***	***p* Value**	**Test**
Laurencia	Seasons	3	35.91	11.97	15.11	<0.0001	One-way Anova
	Residuals	35	27.72	0.79			
	Spring- Autumn		**difference**	**lwr**	**upr**		
Laurencia post-hoc	Summer-Autumn		2.01	0.88	3.14	<0.001	TukeyHSD
	Winter-Autumn		2.65	1.52	3.78	<0.0001	TukeyHSD
	Summer-Spring		1.14	0.08	2.19	0.03	TukeyHSD
	Winter-Spring		0.64	-0.49	1.77	0.44	TukeyHSD
	Winter-Summer		-0.88	-1.93	0.18	0.13	TukeyHSD
			-1.52	-2.57	-0.46	<0.01	TukeyHSD
	**Comparison**	***df***	**Sum Sq**	**Mean Sq**	***F***	***p* Value**	**Test**
Jania	Season	3	2.38	0.79	7.701	<0.001	One-way Anova
	Residuals	32	3.30	0.10			
			**difference**	**lwr**	**upr**		
Jania post-hoc	Spring-Autumn		0.16	-0.25	0.57	0.70	TukeyHSD
	Summer-Autumn		-0.15	-0.56	0.26	0.75	TukeyHSD
	Winter-Autumn		0.54	0.13	0.95	0.01	TukeyHSD
	Summer-Spring		-0.32	-0.73	0.09	0.18	TukeyHSD
	Winter-Spring		0.37	-0.04	0.78	0.08	TukeyHSD
	Winter-Summer		0.69	0.28	1.10	<0.001	TukeyHSD
	**Comparison**	***df***	**Sum Sq**	**Mean Sq**	***F***	***p* Value**	**Test**

Hypnea	Season	3	2.14	0.71	1.825	0.19	Permutation Anova
	Residuals	29	11.34	0.39			
	**Comparison**	***df***	**Sum Sq**	**Mean Sq**	***F***	***p* Value**	**Test**
Padina	Season	3	1216.70	405.58	8.738	<0.001	Permutation Anova
	Residuals	32	1485.30	46.42			
			**t**	**difference**			
Padina post-hoc	Spring-Autumn		1.93	11.32		0.27	Games-Howell
	Summer-Autumn		0.81	7.58		0.85	Games-Howell
	Winter-Autumn		1.84	5.58		0.35	Games-Howell
	Summer-Spring		3.56	17.70		0.01	Games-Howell
	Winter-Spring		5.23	12.93		<0.001	Games-Howell
	Winter-Summer		1.76	11.42		0.34	Games-Howell
	**Comparison**	***df***	**Sum Sq**	**Mean Sq**	***F***	***p* Value**	**Test**
Sargassum	Season	2	4145.00	2072.50	17.68	<0.0001	One-way Anova
	Residuals	18	2110.00	117.20			
	**Comparison**		**difference**	**lwr**	**upr**		
Sargassum post-hoc	Summer-Spring		-10.02	-28.44	8.41	0.37	TukeyHSD
	Winter-Spring		-30.11	-43.14	-17.08	<0.0001	TukeyHSD
	Winter-Summer		-20.09	-38.52	-1.67	0.03	TukeyHSD
	**Comparison**	***df***	**Sum Sq**	**Mean Sq**	***F***	***p* Value**	**Test**
Dictyota	Season	5	198.00	66.01	5.036	<0.01	One-way Anova
	Residuals	26	340.80	13.11			
			**difference**	**lwr**	**upr**		
Dictyota post-hoc	Spring-Autumn		6.75	0.12	13.37	0.04	TukeyHSD
	Summer-Autumn		1.66	-4.96	8.28	0.90	TukeyHSD
	Winter-Autumn		1.21	-5.42	7.83	0.96	TukeyHSD
	Summer-Spring		-5.09	-9.77	-0.40	0.03	TukeyHSD
	Winter-Spring		-5.54	-10.22	-0.86	0.02	TukeyHSD
	Winter-Summer		-0.45	-5.14	4.23	0.99	TukeyHSD
	**Comparison**	***df***	**Sum Sq**	**Mean Sq**	***F***	***p* Value**	**Test**
Ulva	Season	3	5.17	1.72	8.36	<0.01	Permutation Anova
	Residuals	20	4.12	0.21			
			**t**	**difference**			
Ulva post-hoc	Spring-Autumn		4.88	5.04		0.02	Games-Howell
	Summer-Autumn		9.25	8.18		<0.0001	Games-Howell
	Winter-Autumn		11.74	5.17		<0.001	Games-Howell
	Summer-Spring		0.52	8.46		0.95	Games-Howell
	Winter-Spring		0.69	7.17		0.89	Games-Howell

	Winter-Summer		0.21	12.99		0.99	Games-Howell
	**Comparison**	***df***	**Sum Sq**	**Mean Sq**	***F***	***p* Value**	**Test**
Codium	Season	3	1.38	0.46	13.7	<0.0001	One-way Anova
	Residuals	20	0.67	0.03			
			**difference**	**lwr**	**upr**		
Codium post-hoc	Spring-Autumn		0.73	0.37	1.09	<0.0001	TukeyHSD
	Summer-Autumn		0.24	-0.12	0.61	0.27	TukeyHSD
	Winter-Autumn		0.25	-0.09	0.59	0.20	TukeyHSD
	Summer-Spring		-0.48	-0.78	-0.19	<0.001	TukeyHSD
	Winter-Spring		-0.48	-0.75	-0.21	<0.001	TukeyHSD
	Winter-Summer		0.01	-0.26	0.28	0.99	TukeyHSD
	**Comparison**	***df***	**Sum Sq**	**Mean Sq**	***F***	***p* Value**	**Test**
Antioxidant activity species comparison	Seaweed species	7	31311.40	4473.10	109.6	<0.0001	Permutation Anova
	Residuals	235	9590.2	40.8			

Polyphenols (mg PE g^{-1} DW)

**Species**	**Comparison**	***df***	**Sum Sq**	**Mean Sq**	***F***	***p* Value**	**Test**
Padina	Season	3	97.60	32.53	2.49	0.08	One-way Anova
	Residuals	32	418.60	13.08			
	**Comparison**	***df***	**Sum Sq**	**Mean Sq**	***F***	***p* Value**	**Test**
Sargassum	Season	2.00	1616.70	1616.65	23.72	<0.0001	One-way Anova
	Residuals	25.00	1703.80	68.15			
	**Comparison**	***df***	**Sum Sq**	**Mean Sq**	***F***	***p* Value**	**Test**
Dictyota	Season	3	81.94	27.31	7.15	<0.001	Permutation Anova
	Residuals	32	122.20	3.82			
			**t**	**difference**			
Dictyota post-hoc	Spring-Autumn		5.07	6.57		<0.01	TukeyHSD
	Summer-Autumn		3.15	13.75		0.03	TukeyHSD
	Winter-Autumn		10.31	10.05		<0.0001	TukeyHSD
	Summer-Spring		1.29	11.50		0.59	TukeyHSD
	Winter-Spring		8.81	15.67		<0.0001	TukeyHSD
	Winter-Summer		1.35	12.55		0.55	TukeyHSD
	**Comparison**	***df***	**Sum Sq**	**Mean Sq**	***F***	***p* Value**	**Test**
Ulva	Season	3	12.18	4.06	12.14	<0.0001	One-way Anova
	Residuals	32	10.70	0.33			
			**difference**	**lwr**	**upr**		
Ulva post-hoc	Spring-Autumn		1.75	0.71	2.80	<0.001	TukeyHSD
	Summer-Autumn		1.27	0.26	2.28	<0.01	TukeyHSD

	Winter-Autumn		2.10	1.09	3.12	<0.0001	TukeyHSD
	Summer-Spring		-0.48	-1.17	0.21	0.26	TukeyHSD
	Winter-Spring		0.35	-0.34	1.04	0.52	TukeyHSD
	Winter-Summer		0.83	0.19	1.47	<0.01	TukeyHSD
	**Comparison**	***df***	**Sum Sq**	**Mean Sq**	***F***	***p* Value**	**Test**
Codium	Season	3	2.07	0.69	5.84	<0.01	Permutation Anova
	Residuals	32	3.78	0.12			
			**t**	**difference**			
Codium post-hoc	Spring-Autumn		0.22	2.16		0.99	TukeyHSD
	Summer-Autumn		0.08	2.08		0.99	TukeyHSD
	Winter-Autumn		1.04	2.25		0.75	TukeyHSD
	Summer-Spring		0.60	10.19		0.93	TukeyHSD
	Winter-Spring		4.10	17.44		<0.01	TukeyHSD
	Winter-Summer		4.06	21.78		<0.01	TukeyHSD
	**Comparison**	***df***	**Sum Sq**	**Mean Sq**	***F***	***p* Value**	**Test**
Polyphenols species comparison	Seaweed species	4	11120.6	2780.15	113.4	<0.0001	Permutation Anova
	Residuals	166	4069.5	24.52			

Total MAAs (mg g^{-1} DW)

**Species**	**Comparison**	***df***	**Sum Sq**	**Mean Sq**	***F***	***p* Value**	**Test**
Laurencia	Seasons	3	0.19	0.06	2.026	0.13	One-way Anova
	Residuals	32	0.99	0.03			
	**Comparison**	***df***	**Sum Sq**	**Mean Sq**	***F***	***p* Value**	**Test**
Jania	Treatment	3	0.67	0.22	53.8	<0.0001	One-way Anova
	Residuals	32	0.13	0.004			
			**difference**	**lwr**	**upr**		
Jania post-hoc	Spring-Autumn		0.04	-0.04	0.12	0.57	TukeyHSD
	Summer-Autumn		-0.11	-0.19	-0.03	0.01	TukeyHSD
	Winter-Autumn		0.27	0.18	0.35	<0.0001	TukeyHSD
	Summer-Spring		-0.15	-0.23	-0.07	<0.001	TukeyHSD
	Winter-Spring		0.23	0.14	0.31	<0.0001	TukeyHSD
	Winter-Summer		0.37	0.29	0.46	<0.0001	TukeyHSD
	**Comparison**	***df***	**Sum Sq**	**Mean Sq**	***F***	***p* Value**	**Test**
Hypnea	Season	3	0.10	0.03	0.11	0.95	Permutation Anova
	Residuals	32	9.34	0.29			

	Comparison	*df*	Sum Sq	Mean Sq	*F*	*p* Value	Test
MAAs species comparison	Seaweed species	2	33.35	16.68	153.3	<0.0001	Permutation Anova
	Residuals	105	11.42	0.11			
